# High-density genetic map and quantitative trait loci map of skin color in hawthorn (*Crataegus pinnatifida* bge. Var. major N.E.Br.)

**DOI:** 10.3389/fgene.2024.1405604

**Published:** 2024-05-30

**Authors:** Dongsheng Wang, Beibei Cheng, Jijun Zhang

**Affiliations:** ^1^ Engineering Research Center of Chestnut Industry Technology, Ministry of Education, Hebei Normal University of Science and Technology, Qinhuangdao, China; ^2^ College of Horticulture Science and Technology, Hebei Normal University of Science and Technology, Qinhuangdao, China; ^3^ Hebei Key Laboratory of Horticultural Germplasm Excavation and Innovative Utilization, Qinhuangdao, China; ^4^ Hebei Higher Institute Application Technology Research and Development Center of Horticultural Plant Biological Breeding, Qinhuangdao, China

**Keywords:** hawthorn, genetic map, skin color, hue angle, RAD

## Abstract

Fruit skin color is an important trait of the hawthorn tree, which has an important influence on fruit quality. *Crataegus pinnatifida* Bge. var. Major N.E.Br. Is one of the most widely cultivated varieties in China and has a long history of medicinal use. In recent years, it has attracted the attention of the world due to its nutritional and medicinal values. Skin color is the focus of breeders and food processors. At present, skin color-related genes have still not been mapped. In this study, “Shandong Da Mianqiu” (♀, red skin color), “Da Huang Mianzha” (♂, yellow skin color) and 131 F1 hybrids were used to construct genetic map of hawthorn by RAD-seq, and QTL mapping was performed by combining these features with the hue angle and the observed color. In this study, 13,260 SNP was assigned to 17 linkage groups, with an integrated map covering 2,297.75 cM was constructed. A total of 5 QTLs related to hawthorn skin color were detected on LG1, LG3 and LG15. Whether hue angle or pericarp color acts as phenotype for QTL mapping, the candidate genes include *bHLH086*, *WD* repeat regions and *Myb*-like. *bHLH*, *WD* and *Myb* play an important role in the color regulation of Hawthorn skin color. These results lay a solid foundation for QTL mapping and molecular marker-assisted breeding of hawthorn.

## 1 Introduction

Hawthorn (*Crataegus*) is a genus with more than 1,000 species, which is widely distributed in Eurasia and North America (Edwards et al., 2012). *Crataegus pinnatifida* Bge. Var. Major N.E.Br. Is the most widely cultivated in north, northeast and northwest China, with characteristic medicinal and rich nutritional properties (Zhang et al., 2022). *Crataegus pinnatifida* Bge. Var. Major is the origin of cultivation with big fruits (Cuizhi and Spongberg, 2003). Skin color is an important characteristic of the fruit and has a great impact on its economic value. Hawthorn fruits are rich in color; in addition to the commonly cultivated red skin varieties, some cultivars have red, orange, yellow, black, or other skin colors. Breeding fresh food varieties with rich colors has always been an important goal of hawthorn breeding. The genetic basis of hawthorn skin color is extremely complex, and it tends to be regarded as a quantitative trait (Zhang, 2019); however, research on the genetic basis of hawthorn skin color is still lacking. Mining of fruit chromogenic genes will lay a foundation for germplasm innovation and molecular breeding of hawthorn in the future.

It is easy to map related genes by constructing a high-density genetic map and then mining molecular markers closely linked to target traits. Breeders use these markers to screen candidate plants, change phenotypic selection to genotypic selection, and shorten the breeding cycle. However, the relatively insufficient genetic mapping limits the molecular-assisted breeding of hawthorn to some extent. Wang G. et al. (2015) constructed a rough genetic map of hawthorn using SRAP markers, with fewer than 100 markers and an average distance of more than 5 cM. Wang (2016) constructed a genetic map of hawthorn plants by using both SRAP and SSR markers, and the marker distance of male and female parents were 7.05 cM and 6.45 cM, respectively. Because of the low density of these two maps, gene mapping and molecular marker-assisted breeding are limited. Restriction site-associated DNA (RAD) is generated via the reduced-representation genome sequencing (RRGS) method. SNPs can be quickly obtained by high-throughput sequencing of specific restriction endonuclease fragments. RAD can effectively reduce the complexity of the genome, increase the efficiency of data collection, and facilitate the construction of higher-density genetic maps (Davey et al., 2011; Andrews et al., 2016). Zhao et al. (2017) used 2b-RAD to construct the first high-density hawthorn genetic map, it covered 1,551.97 cM with 3,894 SNPs, and the average mark interval was reduced to 0.40 cM. Recently, Shi (2019) also used RAD to construct an integrated map that included 6384 SNP markers, with spanning 2,470.02 cM and an average distance of 0.41 cM.

Several researchers have carried out effective QTL mapping on some characters of hawthorn fruits and leaves. 10 and 14 QTLs related to flavonoid synthesis in leaves were mapped in maternal and paternal map in 2014, respectively (Wang, 2016). Shi (2019) detected 2 QTLs for fruit weight, 6 QTLs for skin firmness and 5 QTLs for flesh brittleness. Zhao (2020) used two maps of hawthorn (Zhao et al., 2017; Shi, 2019) to locate the content of flavonoids in leaves and found 29 and 10 QTLs, respectively. Notably, according to genetic mapping, the skin color of apple, pear, peach, Japanese plum and other fruits has been well mapped (Wu et al., 2014; Sun et al., 2015; Salazar et al., 2017; Shi et al., 2020). However, the skin color of hawthorn fruits is not well known.

In this study, “Shandong Da Mianqiu” (red skin color, female parent), “Da Huang Mianzha” (yellow skin color, male parent) and 131 F1 hybrids were used to construct a high-density genetic map of hawthorn by RAD, and QTL mapping was performed by combining the genetic map with the hue angle of the skin. These results lay a solid foundation for QTL mapping and molecular marker-assisted breeding of hawthorn.

## 2 Materials and methods

### 2.1 Plant material

“Shandong Da Mianqiu” and “Da Huang Mianzha” were selected as the female and male parent, and their fruit skin color are orange and yellow, respectively. F1 seeds were obtained by artificial pollination in 2015, they were sowed in 2016, and 131 F1 plants grew normally at last.

### 2.2 Determination of the hue angle of hawthorn skin

Ten mature fruits were randomly selected from each hawthorn tree. A* and b* were measured by a color difference metre, and the hue angle was calculated based on h° = arctan (b*/a*). Positive a* indicates red-purple; negative a* bluish-green. Positive b* indicates yellow and negative b* blue. For hue angle, 0° represents red and 90° represents yellow (McGuire, 1992). In addition, the fruits pericarp color of parents and offspring were divided into yellow, orange, red and crimson, and were assigned values of 0, 1, two and three respectively for mapping.

### 2.3 DNA extraction and SNP sequencing of hawthorn

The tender leaves of the hawthorn plants were collected, and DNA was extracted with a DNeasy Plant Mini Prep Kit (Qiagen). DNA quality was detected by using Nano Drop 2000 spectrophotometer (Thermo Fisher Scientific, United States), after which DNA was digested with *EcoR* I. P1 adapters (Illumina, United States) were then ligated to the *EcoR* I restriction site of each fragment. Hereafter, adapter-ligated fragments were pooled for random interruption by ultrasonication (ultrasonic interruption at 4°C for 25 s, intermittence for 18 s, 20 cycles). DNA fragments varying from 200 to 500 bp were isolated by 2% agarose gel electrophoresis. 5′and 3′ends of the fragments were flattened with Quick Blunting Kit (NEB), and the samples were purified using a DNA purification kit (Tiangen), then A (adenine) was added on the 3′end at 37°C by Klenow exo- (NEB) (Baird et al., 2008). Subsequently, P2 adapter with part double chains bifurcate into Y-shaped was ligated to these fragments. Both parents and F1 were established in separate libraries. The fragments were used for PCR after purifying. Finally, the prepared RAD libraries were sequenced on an Illumina Hiseq 2,500 platform.

### 2.4 Detection of population SNPs

The raw data were filtered to obtain high-quality clean data, then hawthorn genome assembled by our research group was used as the reference genome for analyzing the sample comparison rate, coverage depth and coverage via BWA software (Li and Durbin, 2009).

First, the Haplotype mode of GATK software (McKenna et al., 2010) was used to detect SNPs. In order to ensure the accuracy of the subsequent analysis, strict data cleaning was carried out according to the following standards: (1) Unless the parent SNP reads support was more than 10 and the quality value ≥ 30, the SNP locus is marked as missing; (2) Filter out the missing population SNP loci in the two parental samples; (3) The reads support number of SNP in the offspring was ≥3 and the quality value was ≥20, otherwise it was marked as missing; (4) The number of parent SNP base supports was more than 10, and the number of F1 SNP base supports was not less than 3; (4) SNP with a deletion rate of more than 20% in 131 offspring samples were filtered out; (5) Individuals with SNP deletion rate greater than 80% were filtered out; (6) Non-binary SNPs are filtered out.

### 2.5 Bin processing and construction of the genetic map

The SNP of the progeny was compared with that of parent. If it was the same as “Da Huang Mianzha,” it was marked as P1. If it was the same as “Shandong Da Mianqiu,” it was marked as P2. If it was missing or heterozygous, it was marked as H (Li et al., 2021; Zhao et al., 2023). Subsequently, LepMap3 was used to analyze the encoded “P1P2H” matrix (Rastas, 2017). Firstly, the markers with segregation distortion were filtered according to *p* < 0.01 by the filtering2 function. Then, the linkage groups were divided by the separatechromosomes2 function of the software, and then the single marker which was not included in the linkage group was re-classified into the linkage group by using the joinsingles2all function. Finally, the genetic distance of each linkage group was calculated by using the ordermarkers2 function.

A sliding window (15 SNPs as a window, sliding 1 SNP each time) was used to find more accurate recombined points. The ratio of different genotypes in each window was calculated, and the genotype of each window was determined according to the majority principle (Qin et al., 2022). Finally, the windows of the same genotype were merged into a recombination bin (Huang et al., 2009).

### 2.6 Mapping and annotation of skin color-related genes

After obtaining the genetic map, combined with the phenotypic information, R/qtl package was used for QTL mapping (Broman et al., 2003). Phenotypic and genotypic data were saved as csv files. Interval mapping was used to identify the QTLs in the R/qtl package. Firstly, genotype probabilities were first calculated, and then Haley-Knott regression analysis (an Interval mapping method) was used to perform QTL genome-wide scanning to obtain the lod value on the entire linkage group. Then, the simulated LOD value of each trait was calculated with 1,000 permutations, and the corresponding LOD value (*p* < 0.05) was selected as the threshold value of this trait. Finally, according to the significance threshold, the loci significantly associated with traits were selected. Estimate 1.5 times LOD interval and 95% Bayes interval and predict QTL location according to significant sites.

The QTL was named with reference to previous methods (Mccouch et al., 1997). The 50 kb region upstream and downstream of the QTL region exceeding the threshold was considered a candidate region, and the NR, SwissProt, EMBL and KEGG databases were used for gene function annotation to screen for regulatory genes related to skin color.

## 3 Results

### 3.1 Analysis of the hue angles of parents and F1 progeny

**FIGURE 1:**
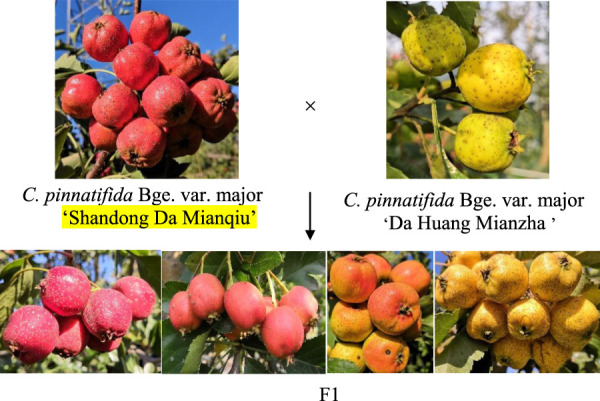
Skin colors classification of parents and offspring (The colors of F1 generations from left to right were crimson, red, orange and yellow respectively).

The skin color of the F1 offspring showed obvious differentiation, which could be divided into crimson, red, yellow and orange by visual observation (Figure 1). The hue angle significantly differed between the two parents and their offspring. The maximum hue angle of F1 was 89.87, which was greater than that of the male parent (81.68), while the minimum hue angle of the F1 generation was 34.06, which was less than the hue angle of the female parent (45.85). The variation coefficient of the hue angle in F1 was 26.03%, and the kurtosis and skewness of the hue angle were −1.43 and −0.36, respectively, indicating a negative offset (Table 1), which was basically in line with the normal distribution. The individuals with hue angles of 10° < h°≤ 40°, 40° < h°≤ 75° and 75° < h°≤ 95° in F1 accounted for 7.41%, 57.41%, and 35.18%, respectively (Supplementary Table S1). Through the color assignment of parents and offspring, it was found that the coefficient of variation in offspring reaches 79.93%.

**TABLE 1:** Statistical analysis of hue angle and pericarp color of parents and F1.

Phenotype	“Da huang Mianzha”	“Shandong Da Mianqiu”	F1					
	Mean ± SD	Mean ± SD	Max	Min	Mean	CV (%)	Kurtosis	Skewness
Hue angle	81.68 ± 0.41	45.85 ± 1.82	89.87	34.06	63.03	26.03	−1.43	−0.36
Pericarp color	0	2	3	0	1.7	79.93	−1.78	−0.28

### 3.2 Analysis of sequencing results

A total of 466.55 Gbp of high-quality data were obtained by RAD sequencing; the male parent and female parent had 3.86 Gbp and 4.80 Gbp, respectively, and the average amount of data from the offspring was 3.50 Gbp. The average Q30 was greater than 92% (Table 2). Furthermore, the sample mapping ratio, genome coverage was analyzed (Table 2). The average mapping ratios of the male parent, female parent and F1 population reached 89.01%, 88.45% and 88.19%, respectively. These results indicated that the integrity of the mutation detection was high. The average coverage (1×) of the male parent, female parent and F1 generation were 20.04%, 18.98% and 19.80%, respectively, which indicated that the accuracy of the mutation detection was good.

**TABLE 2:** Statistics of sequencing data quality and comparison results.

Sample ID	Clean reads (Gb)	Clean Q30 (%)	Mapped ratio (%)	Genome coverage (1×) (%)
Male	3.86	92.67	89.01	20.04
Female	4.80	93.05	88.45	18.98
F1	3.50	92.65	88.19	19.80

### 3.3 Population SNP detection and construction of the genetic map

**FIGURE 2:**
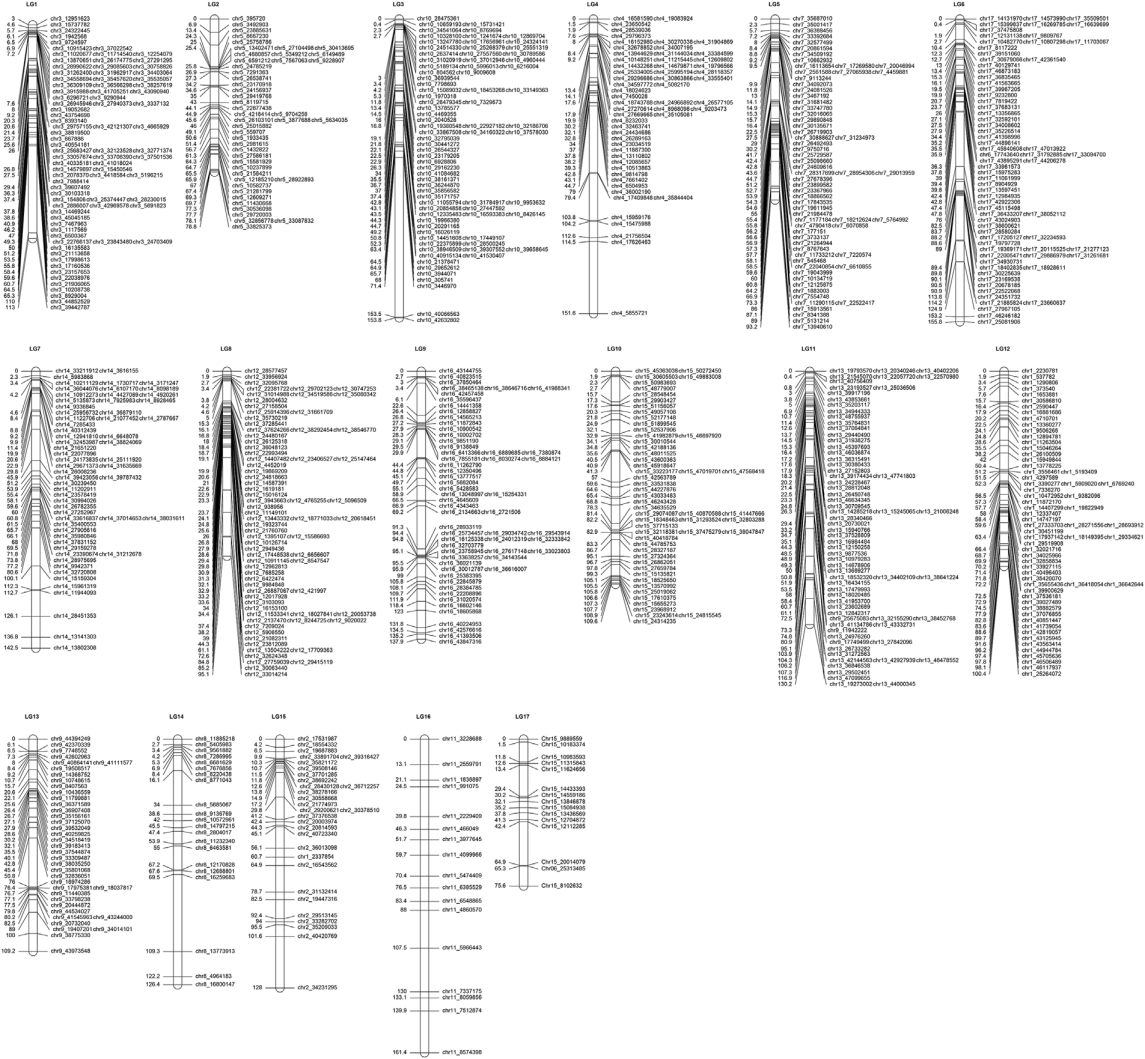
Integrated LGs of hawthorn using the “Shandong Da Mianqiu” and “Da Huang Mianzha” cross (LG1– LG17 are the linkage group numbers of the genetic map).

A total of 7,276,390 original SNPs were detected by GATK, and 13,260 SNP markers were used to construct genetic map. To simplify the computations while ensuring the quality of the map, these SNPs were delimited to 3,026 bins, and they were assigned to the integrated map (Table 3; Figure 2; Supplementary Figure S1). The integrated map included 17 LGs and covered 2,297.75 cM, with average interval distances of 0.85 cM (Table 3). The individual LGs ranged from 98.53 cM (LG8) to 167.3 cM (LG4), with an average length of 135.16 cM. The largest gap varied from 4.6 (LG3) to 15.77 (LG12).

**TABLE 3:** Statistics of integrated linkage map.

Linkage groups	SNP marker	Bin Maker	Distance (cM)	Average distance (cM)	Max gap (cM)
LG1	1768	247	143.19	0.58	5.75
LG2	1,509	161	101.38	0.63	10.06
LG3	1,275	291	161.48	0.55	4.6
LG4	1,151	256	167.3	0.65	8.47
LG5	1,139	208	128.12	0.62	11.26
LG6	1,072	243	157.74	0.65	7.31
LG7	1,054	218	144	0.66	6.92
LG8	788	167	98.53	0.59	5.75
LG9	782	187	139.43	0.75	8.47
LG10	653	178	119.95	0.67	8.47
LG11	598	194	133.3	0.69	6.53
LG12	412	141	129.56	0.92	15.77
LG13	381	152	128.01	0.84	9.66
LG14	179	97	132.17	1.36	5.75
LG15	174	100	132.59	1.33	6.14
LG16	173	98	162.51	1.66	8.87
LG17	152	88	118.49	1.35	8.48
Total	13,260	3,026	2,297.75		
Mean	780	178	135.16	0.85	8.13

### 3.4 QTL mapping of skin color and functional annotation of candidate genes

**FIGURE 3:**
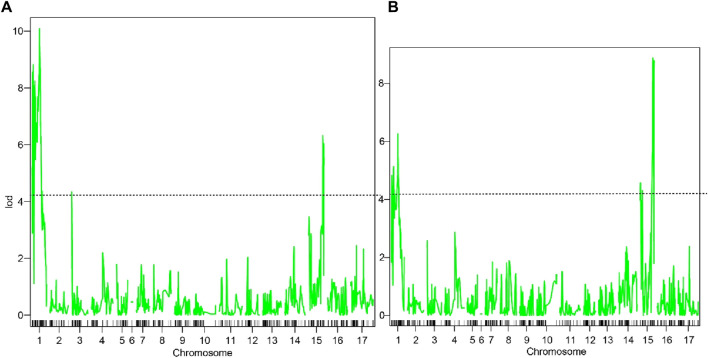
QTL scanning results based on Haley-Knott. **(A)** represented QTL scanning results of hue angle; **(B)** represented QTL scanning results of pericarp color. The dotted line represented the threshold LOD value.

The threshold LOD values (*p* < 0.05) of hue angle and pericarp color was 4.14 and 4.18 based on permutation test, respectively (Figure 3). Three QTLs relating to hue angle were detected, and they were in LG1, LG3 and LG15. Two QTLs of pericarp color were detected, with LOD varying from 6.26 to 8.88 (Table 4).

**TABLE 4:** The QTLs of skin color detected in the F1 population.

Phenotype	Chromosomes	QTL name	Position (cM)	LOD	P-val
Hue angle	1	*qHA-1-1*	51.2	10.08	<0.0001
	3	*qHA-3-1*	0	4.34	0.033
	15	*qHA-15–1*	99	6.32	0.001
Pericarp color	1	*qPC-1-1*	51.2	6.26	<0.0001
	15	*qPC-15–1*	99.3	8.88	<0.0001

Italic values represent the related QTL locus and are named according to Mccouch et al., 1997.

The number of candidate genes that controlled the hue angle and pericarp color was both three (Table 5). It was worth noting that A0A5N5GEA5 and A0A540K9Q7 were annotated in two traits. They were related to transcription factor *bHLH086* and *WD* repeat regions, respectively. For hue angle, a *Myb*-like gene was annotated on chromosome 3, and for pericarp color, a *Myb4*-like gene was also annotated on chromosome 15.

**TABLE 5:** Functional annotation of candidate genes regulating skin color.

Phenotype	Gene name	Chr	Gene start-end (bp)	Functional annotation
Hue angle	A0A5N5GEA5	1	3560885–3562,179	*bHLH086*
	A0A540K9Q7	1	3594025–3597,409	*WD* repeats region domain-containing protein
	A0A5N5FSZ7	3	12922867–12923644	*Myb*-like domain-containing protein
Pericarp color	A0A5N5GEA5	1	3560885–3562,179	*bHLH086*
	A0A540K9Q7	1	3594025–3597,409	*WD* repeats region domain-containing protein
	A0A540NRX3	15	15145238–15147243	myb-related protein *Myb4*-like

Italic values represent the related gene name.

## 4 Discussion

SNP markers are characterized by abundant loci, high density, dimorphism and good stability (Wang et al., 1998) and have obvious advantages in the construction of high-density genetic maps (Wu et al., 2014; Guo et al., 2018). Zhao et al. (2017) used 3894 SNP loci to establish a hawthorn map covering 1,551.97 cM, with an average distance of 0.40 cM. Shi (2019) further used 6,384 SNP markers to construct a genetic map of hawthorns. When there are many SNP markers, the resulting bin map will be more convenient and faster. The bin map uses a certain number of continuous SNPs as the minimum criterion (recombination bin) to determine the occurrence of chromosome recombination, and the probability that the offspring bin originated from the parents is speculated (He et al., 2021). Bin markers not only reduce marker noise but also improve the accuracy of mapping and have great potential in the mapping of complex quantitative traits (Han et al., 2020). However, there is currently no genetic map of hawthorn based on bin molecular markers. In this study, 3,026 bin markers were used to construct an integrated map covering 2,297.75 cM. Which effectively ensures the accuracy of QTL mapping and lays a foundation for mining hawthorn skin color-related genes.

Hawthorn skin has various colors and complex pigment components. Liu et al. (2010) reported that the main pigment component of hawthorn skin was cyanidin 3-O-galactoside. Wang J. et al. (2015) suggested that in addition to cyanidin 3-O-β-galactoside, cyanidin 3-O-α-arabinoside also accounts for a considerable proportion of hawthorn. In a recent study, cyanidin-3-O-glucoside and cyanidin-3-O-galactoside were identified as the main causes of *Crataegus maximowiczii* black skin color (Zhang et al., 2023). However, the anthocyanin compounds present in yellow skin hawthorn fruits have not been well characterized. As a comprehensive color index, the hue angle can reflect the color change in hawthorn skin to a certain extent (Zhao, 2015; Zhang, 2019). Iglesias et al. (1999) reported that there was a linear relationship between the anthocyanin content and hue angle in the skin of “Delicious” apple plants. The higher the anthocyanin content is, the smaller the hue angle. In general, 10° < *h*°≤ 40°, 40° < *h*°≤ 75° and 75° < *h*°≤ 95° represent red, orange, and yellow peel, respectively (Li et al., 2016). The hue angle of parents and F1 was consistent with the color performance observed directly. The color of plum skin is also consistent with the hue angle, and the hue angle can be used as an important index of color (Zhou et al., 2015). The hue angle of hawthorn plants exhibited the same pattern of inheritance as that of many other fruits. Volz et al. (2008) suggested that the interaction of at least two dominant genes controls the inheritance of “Huobali” pear red skin. Four QTLs for the red skin of “Bayuehong” pear were detected and located on LG4, LG13 and LG16 (Wu et al., 2014). Hue angle was used as the phenotypic index of skin color, and a total of 5 QTLs were found in this study. These segments are located on LG1, LG3and LG15, respectively.

Because the anthocyanin metabolic pathway is highly conserved (Winkel-Shirley, 2001), the transcription factors involved in anthocyanin metabolism in other species are likely to be involved in the regulation of hawthorn skin color. A total of four transcription factors were detected, including 2 *MYB*, one *bHLH*, 1 *WD*. *MYB*, *bHLH,* and *WD40* have been proven to play an individual or combined role in regulation of skin color of many fruit trees. *MdMYB1* and *MdMYB10* were first proven to be genes that regulate skin color in apple plants, especially in terms of controlling the presence of red pigments (Takos et al., 2006; Espley et al., 2007; McClure et al., 2019). In addition, it is believed that the highly variable *MYB10* gene is the main regulatory gene involved in Japanese plum fruit skin color variation (Fiol et al., 2021). *MdMYB3* was subsequently reported to promote anthocyanin accumulation in apple skin (Vimolmangkang et al., 2013). Chen et al. (2022) found that *bHLH*s were closely related to plum fruit color through transcriptomic and metabolic analysis. *BHLH, especially MdbHLH3 and MdbHLH33,* is also involved in the synthesis of apple anthocyanins to a great extent (Xie et al., 2012; Xu et al., 2017). Additionally, *WD*40 can interact with *bHLH* TFs to increase anthocyanin accumulation in apple plants (An et al., 2012). In fact, as a major regulatory element, MBW (*MYB*-*bHLH*-*WD40*) complex interact with each other to affect the expression of structural genes in anthocyanin synthesis, resulting in differences in color patterns (Stracke et al., 2001; Feller et al., 2011; Wang et al., 2013).

## 5 Conclusion

In this study, 13,260 SNP was assigned to 17 linkage groups. An integrated map covering 2,297.75 cM was constructed. A total of 5 QTLs related to hawthorn skin color were detected on LG1, LG3 and LG15. Whether hue angle or pericarp color acts as phenotype for QTL mapping, the candidate genes include *bHLH086*, *WD* repeat regions and *Myb*-like. Thus, it is speculated that *bHLH*, *WD,* and *Myb* play an important role in the color regulation of Hawthorn skin color. This study has made a useful contribution to the high-density genetic map of hawthorn, and it is also useful to explore the regulatory mechanism of hawthorn skin color and promote molecular-assisted breeding.

## Data Availability

The data presented in the study are deposited in the NCBI repository, accession number PRJNA1029972: https://www.ncbi.nlm.nih.gov/bioproject/PRJNA1029972.
