# Supplementary material for: High-density genetic map and quantitative trait loci map of skin color in hawthorn (Crataegus pinnatifida bge. Var. major N.E.Br.)
Source: Front Genet. 2024 May 30;15:1405604. doi: 10.3389/fgene.2024.1405604 (PMC11169616; doi:10.3389/fgene.2024.1405604)
Supplement: Supplementary file 1 [file Table1.docx]

Supplementary Material

# Supplementary Figures and Tables

## Supplementary Figures


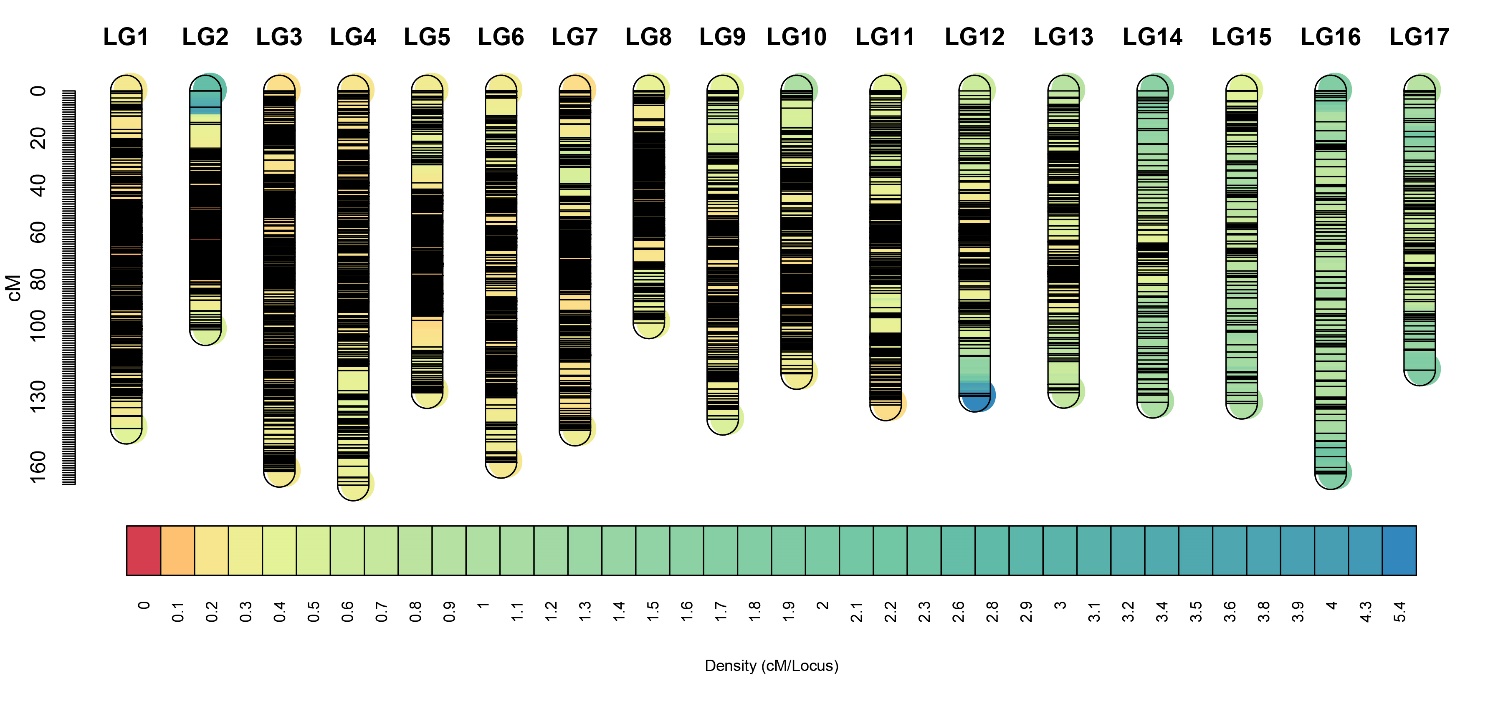


**Supplementary Figure 1** Markers distribution of integrated Linkage groups

## Supplementary Tables

**Supplementary table 1.** Measurement data of hue angle and pericarp color.

| Samples | pericarp color | hue angle | Samples | pericarp color | hue angle |
| --- | --- | --- | --- | --- | --- |
| Female | 2 | 45.85 | 66 | * | * |
| Male | 0 | 81.68 | 67 | 0 | 86.47 |
| 1 | 3 | 48.64 | 68 | 0 | 83.54 |
| 2 | 3 | 39.74 | 69 | * | * |
| 3 | 3 | 46.17 | 70 | 3 | 48.03 |
| 4 | 3 | 73.99 | 71 | 0 | 80.79 |
| 5 | 0 | 83.16 | 72 | 3 | 51.36 |
| 6 | 0 | 81.25 | 73 | 3 | 36.95 |
| 7 | 0 | 81.11 | 74 | 2 | 52.33 |
| 8 | 0 | 89.03 | 75 | * | * |
| 9 | 0 | 81.98 | 76 | 1 | 78.09 |
| 10 | 3 | 54.00 | 77 | 1 | 68.18 |
| 11 | 3 | 48.40 | 78 | 3 | 44.37 |
| 12 | 2 | 64.78 | 79 | 0 | 80.96 |
| 13 | 3 | 51.24 | 80 | 0 | 80.88 |
| 14 | 3 | 82.44 | 81 | 1 | 60.17 |
| 15 | 2 | 63.37 | 82 | 1 | 55.02 |
| 16 | 3 | 87.04 | 83 | 3 | 44.58 |
| 17 | 0 | 84.16 | 84 | 2 | 34.61 |
| 18 | 0 | 72.65 | 85 | 3 | 39.91 |
| 19 | 3 | 82.30 | 86 | 0 | 80.57 |
| 20 | 0 | 86.49 | 87 | 3 | 54.55 |
| 21 | * | * | 88 | 0 | 78.36 |
| 22 | 0 | 54.76 | 89 | 0 | 83.78 |
| 23 | 3 | 42.85 | 90 | 3 | 51.65 |
| 24 | 3 | 57.25 | 91 | 3 | 48.22 |
| 25 | 0 | 89.87 | 92 | 3 | 40.98 |
| 26 | 3 | 38.18 | 93 | 0 | 73.60 |
| 27 | 0 | 85.66 | 94 | 3 | 44.37 |
| 28 | 3 | 42.29 | 95 | 3 | 35.28 |
| 29 | * | * | 96 | 0 | 82.15 |
| 30 | 3 | 43.39 | 97 | * | * |
| 31 | 3 | 34.06 | 98 | 0 | 77.36 |
| 32 | 3 | 43.18 | 99 | 0 | 75.78 |
| 33 | 2 | 66.97 | 100 | * | * |
| 34 | * | * | 101 | * | * |
| 35 | 0 | 69.32 | 102 | * | * |
| 36 | 0 | 78.73 | 103 | * | * |
| 37 | 1 | 64.44 | 104 | * | * |
| 38 | * | * | 105 | * | * |
| 39 | 0 | 55.45 | 106 | 3 | 53.41 |
| 40 | * | * | 107 | * | * |
| 41 | 2 | 75.27 | 108 | 2 | 77.88 |
| 42 | 2 | 54.93 | 109 | 0 | 77.90 |
| 43 | * | * | 110 | 0 | 73.08 |
| 44 | 0 | 78.14 | 111 | 3 | 54.61 |
| 45 | 3 | 49.25 | 112 | 0 | 69.36 |
| 46 | 3 | 44.30 | 113 | 3 | 40.71 |
| 47 | 2 | 59.79 | 114 | 3 | 46.81 |
| 48 | 0 | 81.60 | 115 | 3 | 59.47 |
| 49 | 3 | 56.58 | 116 | 3 | 53.10 |
| 50 | * | * | 117 | 0 | 84.50 |
| 51 | 0 | 85.81 | 118 | * | * |
| 52 | 3 | 45.28 | 119 | 3 | 57.40 |
| 53 | 2 | 64.89 | 120 | 1 | 78.80 |
| 54 | * | * | 121 | 0 | 70.28 |
| 55 | 3 | 62.98 | 122 | 1 | 84.80 |
| 56 | * | * | 123 | 3 | 40.56 |
| 57 | 3 | 67.69 | 124 | 3 | 83.02 |
| 58 | * | * | 125 | 0 | 69.99 |
| 59 | * | * | 126 | 3 | 45.92 |
| 60 | 1 | 77.68 | 127 | 3 | 46.72 |
| 61 | 3 | 48.83 | 128 | 0 | 79.46 |
| 62 | 1 | 56.78 | 129 | 3 | 41.52 |
| 63 | 3 | 39.28 | 130 | 0 | 81.14 |
| 64 | 3 | 55.05 | 131 | 3 | 51.01 |
| 65 | 3 | 62.31 |  |  |  |

Note: ‘*’ indicates that hue angle or pericarp color has not been measured, due to there is no fruit.
