# Supplementary figures and images for: High-density genetic map and quantitative trait loci map of skin color in hawthorn (Crataegus pinnatifida bge. Var. major N.E.Br.)
Source: Front Genet. 2024 May 30;15:1405604. doi: 10.3389/fgene.2024.1405604 (PMC11169616; doi:10.3389/fgene.2024.1405604)

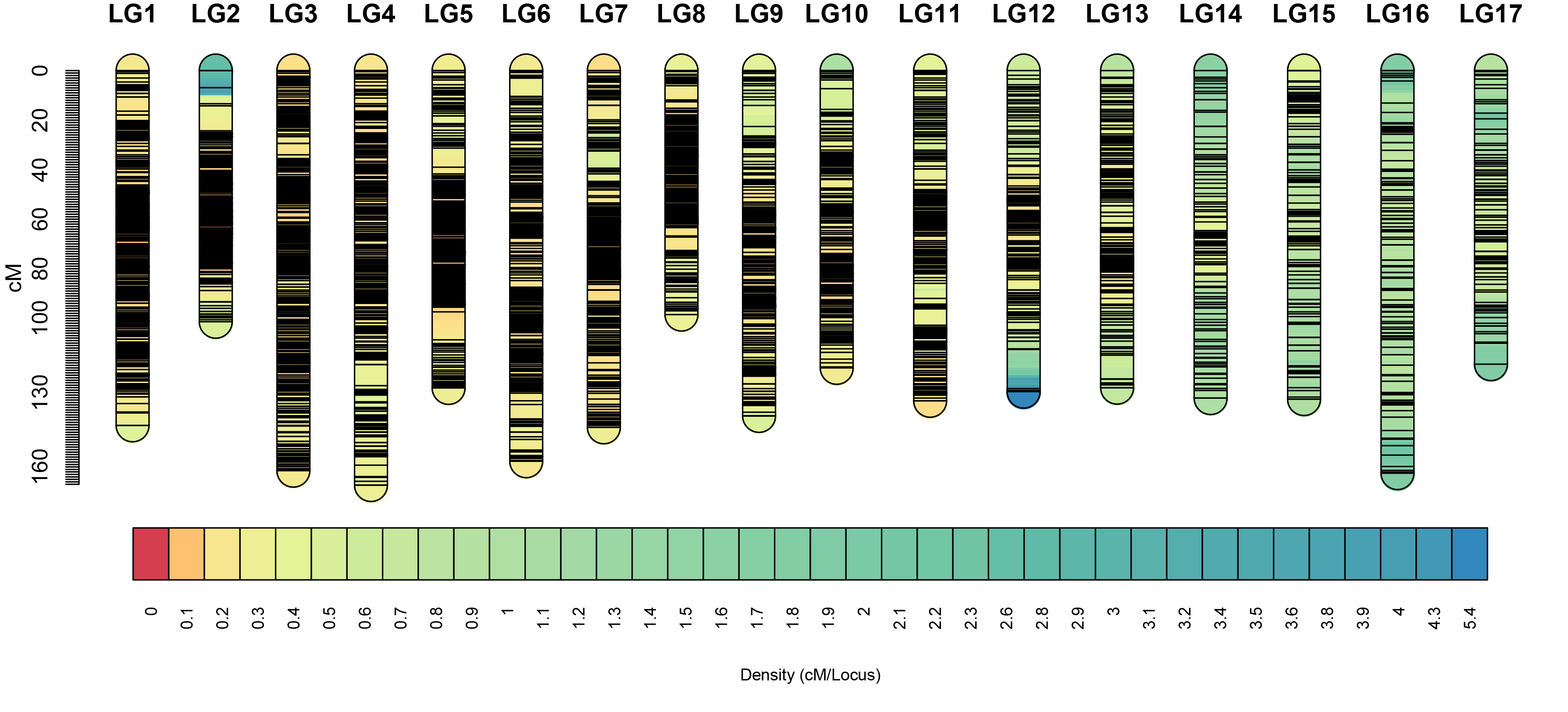

Supplement: Supplementary file 2 [file Image1.JPEG]
